# COVID-19 Pandemic as an Equalizer of the Health Returns of Educational Attainment for Black and White Americans

**DOI:** 10.1007/s40615-023-01601-w

**Published:** 2023-07-25

**Authors:** Arash Rahmani, Babak Najand, Najmeh Maharlouei, Hossein Zare, Shervin Assari

**Affiliations:** 1 Marginalized-Related Diminished Returns (MDRs) Center, Los Angeles, CA USA; 2 Department of Health Policy and Management, Johns Hopkins Bloomberg School of Public Health, Baltimore, MD 21205 USA; 3 School of Business, University of Maryland Global Campus (UMGC), Adelphi, 20783 USA; 4 Department of Family Medicine, Charles R Drew University of Medicine and Science, Los Angeles, CA USA; 5 Department of Urban Public Health, Charles R Drew University of Medicine and Science, Los Angeles, CA USA

**Keywords:** Socioeconomic status, Educational attainment, Marginalized-related diminished return, COVID-19 pandemic

## Abstract

**Background:**

COVID-19 pandemic has immensely impacted the social and personal lives of individuals around the globe. Marginalized-related diminished returns (MDRs) theory suggests that educational attainment shows a weaker protective effect for health and behavioral outcomes for Black individuals compared to White individuals. Previous studies conducted before the COVID-19 pandemic demonstrated diminished returns of educational attainment for Black individuals compared to White individuals.

**Objectives:**

The study has three objectives: First, to test the association between educational attainment and cigarette smoking, e-cigarette vaping, presence of chronic medical conditions (CMC), self-rated health (SRH), depressive symptoms, and obesity; second, to explore racial differences in these associations in the USA during the COVID-19 pandemic; and third, to compare the interaction of race and return of educational attainment pre- and post-COVID-19 pandemic.

**Methods:**

This study utilized data from the Health Information National Trends Survey (HINTS) 2020. Total sample included 1313 adult American; among them, 77.4% (*n* = 1017) were non-Hispanic White, and 22.6% (*n* = 296) were non-Hispanic Black. Educational attainment was the independent variable operationalized as years of education. The main outcomes were cigarette smoking, e-cigarette vaping, CMC, SRH, depressive symptoms, and obesity. Age, gender, and baseline physical health were covariates. Race/ethnicity was an effect modifier.

**Results:**

Educational attainment was significantly associated with lower CMC, SRH, depressive symptoms, obesity, cigarette smoking, and e-cigarette vaping. Educational attainment did not show a significant interaction with race on any of our outcomes, suggesting that the health returns of education is similar between non-Hispanic White and non-Hispanic Black individuals.

**Conclusion:**

COVID-19 may have operated as an equalizer of the returns of educational attainment. This observation may be because White may have more to lose; Black communities may be more resilient or have economic and social policies that buffered unemployment and poverty regardless of historical anti-Black oppression.

## Introduction

Socioeconomic status (SES) indicators affect health at the individual and population levels [1–6]. The relation between educational attainment, as a major indicator of socioeconomic status (SES), and health has been investigated in detail [3, 7–9]. High educational attainment has been associated with better health [10] and more desirable health behaviors [11, 12], and low educational attainment is associated with worse health consequences [13]. Furthermore, educational attainment as a primary SES indicator demonstrated protective effects for mental health [14], obesity [15], and smoking [16].

There are significant disparities in health and behavioral outcomes between racial/ethical groups in the USA [17, 18]. For example, although the prevalence of obesity has increased in the general population [19], various racial groups have experienced different increase rates, with non-Hispanic Black individuals experiencing the greatest increase across almost all gender and age groups [20]. Besides, health behaviors such as smoking are not equally distributed among racial groups in the USA [21]. Considering the significance of educational attainment for individuals’ health and racial disparities in health and behavioral outcomes in the USA, more research is needed to elucidate the complex interaction effects of race and educational attainment on important health and behavioral outcomes [22, 23] as well as the dynamics of these effects during considerable societal changes or governmental interventions. Investigation of these dynamics can give a window into the forces that are effective on racial health disparities. Studying specific changes during a definite period of macro-level societal disruptions such as the COVID-19 pandemic (e.g., disruption of the free market, distribution of governmental monetary aids) and their subsequent impacts on differential racial health returns to educational attainment can also have potential practical implications. We can develop policies to eliminate racial disparities in health by taking advantageous and disadvantageous factors identified during COVID-19 (depending on their respective effects on racial health return to educational attainment) into account.

Marginalized-related diminished returns (MDRs) theory [24] is conceptualized as the relative disadvantage of sidelined racial groups in gaining health and financial benefits from their SES resources [25]. Consistent with MDRs, previous research has repeatedly shown that higher educational attainment improves White individuals’ health and behavioral outcomes to a greater extent than Black individuals. In fact, educational attainment demonstrated a weaker protective effect against current smoking [26], obesity [27], and subjective health [28] for Black individuals than White individuals. One possible explanation for such differential interaction of race and SES indicators on health and behavioral outcomes is that socially privileged racial groups can mobilize their resources to gain tangible health benefits or adopt healthy behaviors [29]. That is why SES indicators have a weaker effect in health and behavioral outcomes in a marginalized racial group [27, 30–32]. Under normal social circumstances, it is difficult or even impractical to do a social experiment at a national level on MDRs. Most of the research in this field does not assess any specific government intervention, policy, or significant social period. Hence, unprecedented chaotic social changes during the COVID-19 pandemic looks like an unfrequented chance to study MDRs pattern in a short period of radical socioeconomic changes. We considered COVID a unique opportunity to discover what changes would happen in MDRs (in which direction) and what would be the potential driving forces behind these changes.

The COVID-19 pandemic has had immense social and global impacts [33]. Only in the USA, COVID-19 was responsible for 600,000 excess deaths during the first year of the pandemic [34], causing even higher death rates in underserved racial groups [35]. Previous research showed how the COVID-19 pandemic changed our economy [36], physical health [37], and mental health [38] in drastic ways. The COVID-19 pandemic interrupted patients’ access to healthcare, leading to poor outcomes such as worsening blood pressure (BP) [39]. Regarding health returns of SES, one recent study demonstrated the diminished return of employment in Black individuals during the COVID-19 pandemic [40]. Therefore, considering the effects of the COVID-19 pandemic, assessment of the change in differential returns of educational attainment is of great importance [40, 41].

Two competing hypotheses can be postulated about the impact of macro events such as the COVID-19 pandemic on racialized returns of educational attainment on health (e.g., psychological distress, obesity) and behavioral (e.g., cigarette smoking) outcomes. One hypothesis is that the COVID-19 pandemic may have exacerbated the racial disparities in the return of educational attainment, as it deepened the racial inequalities in the USA in general [42]. There are supporting evidence that the COVID-19 pandemic hit the underserved racial population harder: crowded and high-poverty neighborhood contributed to higher seropositivity among Black and Latino individuals compared to White individuals [43] and Black workers were overrepresented in occupations with high potential risk of exposure to the COVID-19 disease [44]. Based on this hypothesis, the COVID-19 pandemic would maintain or increase the diminished return of the educational attainment for Black individuals — The COVID-19 pandemic is an *Exacerbator.* On the other hand, some findings support equalizing effect of macro events on racial disparities from the COVID-19 pandemic and previous national or social disasters. For example, one recent study showed better mental health outcomes for Black individuals compared to White individuals during the COVID-19 pandemic [45]. In addition, prior to the COVID-19 pandemic, Black and White individuals showed almost similar rates of suicide in the 2009 financial crisis [46] and mental health outcomes during the September 11 Attacks [47]. Consistent with these paradoxically better-than-expected outcomes of Black individuals during macro events, the second hypothesis suggests that COVID-19 pandemic could be considered an equalizing factor for the return of educational attainment — The COVID-19 pandemic is an *Equalizer*, at least for some health and behavioral outcomes or in specific gender and race subgroups [48, 49].

Opposing data from different studies (with different settings, study designs, and outcomes) highlights the necessity for comparing the same parametric between the pre- and post-COVID-19 data. This line of research will help us to determine whether the COVID-19 pandemic had an *Equalize*r or *Exacerbator* in any specific circumstances of interest and to speculate about mechanisms behind such aggravation or alleviation. Understanding whether major social events such as the COVID-19 pandemic strengthen racial disparities or weaken them can have practical implications beyond the COVID-19 pandemic [40, 50, 51].

This study investigated the racial variation in the association of educational attainment with chronic medical conditions (CMC), self-rated health (SRH), depressive symptoms, obesity, cigarette smoking, and e-cigarette vaping among American adults in the USA during the COVID-19 pandemic. This study included these five unrelated outcomes to reflect systemic causes of health inequalities [52, 53]. We intentionally focused on outcomes we would not necessarily expect to be directly related to each other or COVID-19 pandemic.This study pursued three aims. First, we wanted to assess the association between educational attainment and CMC, SRH, depressive symptoms, obesity, cigarette smoking, and e-cigarette vaping. Second, we wanted to compare these associations by race to examine whether we could replicate previously shown diminished return of educational attainment for Black individuals compared to White individuals during the COVID-19 pandemic. Third, we aimed to compare MDRs during the COVID-19 pandemic with previously studied patterns of return of educational attainment among different racial groups in the USA.

## Method

### Design and Settings

This is a cross-sectional secondary analysis of data from the Health Information National Trends Survey (HINTS 2020) study Cycle 4. HINTS 2020 Cycle 4 was done between February 24, 2020, and June 15, 2020. Our secondary analysis was exempt from a full ethics review because we used de-identified data.

### Data Collection

The HINTS 2020 Cycle 4 employed a multi-stage stratified random sampling. For first stage, the sampling addresses were categorized as the following two sampling strata: (1) addresses in regions with low concentrations of minority populations and (2) addresses in regions with high concentrations of minority populations. In the second sampling stage, only one participant was selected from each target household, based on eligibility.

### Sample and Sampling

Adult Americans participated in the HINTS study across US states in 2020. Random samples of addresses were generated by using a database of addresses used by Marketing System Group (MSG). Any non-vacant US residential addresses were entered the sampling frame for HINTS 2020 Cycle 4 including post office (P.O.) boxes, seasonal addresses, and throwbacks. A total number of 3865 individuals completed surveys which resulted in a 37% response rate. However, for this analysis, we included 1313 adult individuals including 1017 non-Hispanic White and 296 non-Hispanic Black who provided complete data on our study variables. We recruited only participants who entered HINTS 2020 Cycle 4 after the COVID-19 pandemic was announced by the Centers for Diseases Control and Prevention (CDC) and the World Health Organization (WHO). Participants were eligible if they were adult (age 18 years or more) and US resident. This study excluded participants who were institutionalized in a correctional or health setting.

## Measures

### Independent Variable

#### Educational Attainment

Highest level of education at the individual level, measured by self-reported question, was our independent variable. The specific question was “ What is the highest grade or level of schooling you completed?” This categorical variable has the following seven categories: (1) Less than 8 years, (2) 8 through 11 years, (3) 12 years or completed high school, (4) Post high school training other than college (vocational), (5) Some college, (6) College graduate, and (7) Postgraduate.

### Dependent Variable

#### Chronic Medical Conditions

We defined the presence of any conditions, including diabetes mellitus (DM), hypertension (HTN), coronary artery disease (CAD), and chronic lung disease, as CMC positive (score = 1) and no condition as CMC negative (score = 0).

#### Self-rated Health

The poor subjective SRH was measured by the conventional SRH item: “In general, would you say your health is …” Item responses can be selected from excellent, very good, good, fair, or poor. We defined excellent, very good, good, and fair as good SRH (score = 0) and the answer “poor” as poor SRH (score = 1). Therefore, our outcome mainly indicated poor not good health.

#### Obesity

We calculated participants’ body mass index (BMI) using self-reported weight and height in pounds and foot/inch. Using the CDC definition [54], BMI equal to or larger than 30 was defined as obesity.

#### Cigarette smoking

We coded 1 for current users and 0 for never or ex-user using a single item.

#### E-cigarette vaping

We coded 1 for current users and 0 for never or ex-users using a single item.

#### Depression

We used PHQ-4 with scores ranging from 0 to 12. Scores higher than 6 were considered depressed and coded as 1.

### Moderators

#### Race

All participants self-identified their race as if they were Non-Hispanic White, Non-Hispanic Black, or from other racial groups. In this study, race was Black/African American (= 1) or White (= 0). We excluded all participants who were from other racial backgrounds.

### Confounders

#### Age

Age was a continuous variable in years self-reported by participants.

#### Gender

Gender, as a dichotomous variable, was self-reported as 1 (if male) 0 (if female) by participants.

#### Marital Status

The participant reported marital status coded as 1 (if married) and 0 for any other status.

### Analysis

For descriptive statistics, we used mean and proportion. For multivariable analysis, we used two logistic regression models: Model 1 did not include race by educational attainment interaction terms. Model 2 included race by educational attainment interaction term. For all models, we used age, gender, and marital status as covariates, and race was the focal moderator. Both logistic regression models were estimated in the pooled sample that included Black and White individuals. Adjusted odds ratios (ORs), 95% confidence intervals (CI), and *p*-values were reported. We applied Statistical Package for the Social Sciences (SPSS) 23.0 (IBM Inc, NY, USA) to perform data analysis.

## Result

### Descriptive Statistics

Table 1 shows the descriptive data for the total sample and for each race. For the total sample (*n* = 1313), the mean age was 55.67 years (SD = 17.64), 77.4% (*n* = 1017) were Non-Hispanic White, and 22.6% (*n* = 296) were Non-Hispanic Black. Non-Hispanic Black individuals had lower educational attainment compared to Non-Hispanic White individuals. White individuals had significantly lower percentage of CMC, poor SRH, and obesity.

**Table 1:** Frequency (%) and mean (SD) of descriptive data overall and by race

	All (*N* = 1313)		Non-Hispanic White (*N* = 1017)		Non-Hispanic Black (*N* = 296)		*P* value
	No	%	No	%	No	%	
CMC							
No	489	37.2	411	40.4	78	26.4	*
Yes	824	62.8	606	59.6	218	73.6	
SRH							
Good	1123	85.5	892	87.7	231	78.0	*
Poor	190	14.5	125	12.3	65	22.0	
Obesity							
No	834	63.5	677	66.6	157	53.0	*
Yes	479	36.5	340	33.4	139	47.0	
PHQ (depressed)							
No	1183	90.1	916	90.1	267	90.2	
Yes	130	9.9	101	9.9	29	9.8	
Cigarette smoking (current)							
No	1152	87.7	898	88.3	254	85.8	
Yes	161	12.3	119	11.7	42	14.2	
E-cigarette vaping (current)							
No	1274	97.0	978	96.2	296	100.0	*
Yes	39	3.0	39	3.8	0	0	
	Mean	SD	Mean	SD	Mean	SD	
Age (y)	55.67	17.644	56.03	17.446	54.44	18.286	*
Education (1–7)	5.1330	1.50715	5.2136	1.49240	4.8527	1.52715	*

*Abbreviatioins: CMC* chronic medical conditions, *SRC* self-rated health, *PHQ* patient health questionnaire

*Significant comparison of White and Black participants (independent t test or chi-square)

As Table 2 shows, educational attainment was significantly associated lower CMC, SRH, depressive symptoms, obesity, cigarette smoking, and e-cigarette vaping. Educational attainment did not show a significant interaction with race on any of our outcomes, suggesting that the health returns of education is similar between White and Black individuals.

**Table 2:** Overall models in the absence and presence of interaction term

	*B*	SE	OR	Lower bound	Upper bound	*P* value	*B*	SE	OR	Lower bound	Upper bound	*P* value
	*Main effects*						*Interaction*					
*CMC*												
BNH (ref. WNH)	0.670	0.159	1.954	1.432	2.666	< 0.001	0.324	0.549	1.382	0.471	4.058	0.556
Male	− 0.198	0.059	0.820	0.730	0.921	0.001	− 0.198	0.059	0.820	0.731	0.921	0.001
Age	0.043	0.004	1.044	1.036	1.052	< 0.001	0.043	0.004	1.044	1.036	1.052	< 0.001
Married	0.021	0.125	1.021	0.799	1.304	0.870	0.019	0.125	1.019	0.798	1.302	0.880
Education (1–7)	− 0.153	0.043	0.858	0.789	0.934	< 0.001	− 0.167	0.049	0.846	0.769	0.931	0.001
Race × education (1–7)	-	-	-	-	-	-	0.068	0.104	1.071	0.873	1.312	0.512
*SRH*												
Race	0.556	0.180	1.743	1.224	2.481	0.002	− 0.482	0.554	0.618	0.209	1.829	0.384
Male	− 0.122	0.075	0.886	0.764	1.027	0.107	− 0.117	0.075	0.890	0.768	1.031	0.120
Age	0.022	0.005	1.022	1.012	1.032	< 0.001	0.021	0.005	1.021	1.011	1.031	< 0.001
Married	− 0.339	0.168	0.712	0.512	0.990	0.043	− 0.343	0.168	0.710	0.511	0.987	0.041
Education (1–7)	− 0.303	0.053	0.739	0.666	0.819	< 0.001	− 0.371	0.063	0.690	0.609	0.781	< 0.001
Race × education (1–7)	-	-	-	-	-	-	0.227	0.114	1.255	1.005	1.569	0.045
*PHQ4*												
Race	− 0.198	0.230	0.821	0.523	1.287	0.389	− 1.224	0.709	0.294	0.073	1.180	0.084
Male	0.072	0.077	1.075	0.924	1.251	0.348	0.075	0.077	1.077	0.926	1.254	0.334
Age	− 0.017	0.005	0.983	0.973	0.993	0.001	− 0.018	0.005	0.982	0.972	0.992	0.001
Married	− 0.400	0.194	0.670	0.458	0.980	0.039	− 0.402	0.194	0.669	0.458	0.978	0.038
Education (1–7)	− 0.258	0.061	0.772	0.686	0.870	< 0.001	− 0.309	0.069	0.734	0.642	0.840	< 0.001
Race × education (1–7)	-	-	-	-	-	-	0.224	0.143	1.251	0.945	1.656	0.117
*Obesity*												
Race	0.546	0.138	1.727	1.319	2.261	< 0.001	0.706	0.462	2.026	0.820	5.008	0.126
Male	0.114	0.062	1.121	0.993	1.265	0.065	0.114	0.062	1.121	0.992	1.265	0.066
Age	− 0.004	0.004	0.996	0.989	1.003	0.250	− 0.004	0.004	0.996	0.989	1.003	0.259
Married	0.010	0.118	1.010	0.802	1.274	0.930	0.011	0.118	1.011	0.802	1.274	0.927
Education (1–7)	− 0.103	0.039	0.902	0.835	0.974	0.009	− 0.095	0.045	0.910	0.833	0.994	0.036
Race × education (1–7)	-	-	-	-	-	-	− 0.032	0.089	0.968	0.812	1.154	0.717
*Cigarette smoking*												
Race	0.012	0.202	1.012	0.681	1.504	0.952	− 0.153	0.595	0.858	0.267	2.756	0.797
Male	0.136	0.084	1.146	0.971	1.352	0.107	0.136	0.084	1.146	0.971	1.352	0.106
Age	− 0.012	0.005	0.988	0.978	0.997	0.012	− 0.012	0.005	0.988	0.978	0.997	0.011
Married	− 0.582	0.180	0.559	0.392	0.795	0.001	− 0.582	0.180	0.559	0.392	0.795	0.001
Education (1–7)	− 0.348	0.056	0.706	0.633	0.788	< 0.001	− 0.357	0.064	0.700	0.617	0.794	< 0.001
Race × education (1–7)	-	-	-	-	-	-	0.038	0.127	1.038	0.809	1.332	0.767
*E-cigarette vaping*												
Race	− 18.378	2149.630	0.000	0.000	0.000	0.993	− 20.118	6926.400	0.000	0.000	0.000	0.998
Male	0.309	0.140	1.362	1.036	1.791	0.027	0.309	0.140	1.362	1.036	1.791	0.027
Age	− 0.052	0.009	0.950	0.932	0.967	< 0.001	− 0.052	0.009	0.950	0.932	0.967	0.000
Married	− 0.739	0.358	0.477	0.237	0.962	0.039	− 0.739	0.358	0.477	0.237	0.962	0.039
Education (1–7)	− 0.400	0.111	0.670	0.540	0.832	< 0.001	− 0.400	0.111	0.670	0.540	0.832	< 0.001
Race × education (1–7)	-	-	-	-	-	-	0.393	1353.115	1.482	0.000	0.000	1.000

*Abbreviatioins: CMC* chronic medical conditions, *SRC* self-rated health, *PHQ4* patient health questionnaire.

As Table 3 shows, educational attainment was similarly associated with CMC, SRH, depressive symptoms, obesity, and cigarette smoking in White and Black individuals.

**Table 3:** Race-stratified models

	*B*	SE	OR	Lower bound	Upper bound	*P* value	*B*	SE	OR	Lower bound	Upper bound	*P* value
	Non-Hispanic White						Non-Hispanic Black					
CMC												
Male	− 0.162	0.072	0.850	0.739	0.979	0.024	− 0.303	0.113	0.738	0.591	0.922	0.008
Age	0.041	0.004	1.042	1.033	1.051	< 0.001	0.052	0.010	1.053	1.034	1.073	< 0.001
Married	− 0.009	0.138	0.991	0.757	1.297	0.946	0.136	0.302	1.146	0.635	2.070	0.651
Education (1–7)	− 0.170	0.048	0.844	0.767	0.928	< 0.001	− 0.098	0.095	0.906	0.752	1.092	0.301
SRH												
Male	− 0.076	0.103	0.927	0.758	1.134	0.462	− 0.160	0.117	0.853	0.678	1.073	0.173
Age	0.024	0.006	1.024	1.011	1.037	< 0.001	0.015	0.009	1.015	0.997	1.034	0.101
Married	− 0.458	0.200	0.633	0.427	0.937	0.022	− 0.042	0.306	0.959	0.527	1.747	0.892
Education (1–7)	− 0.368	0.064	0.692	0.611	0.784	< 0.001	− 0.150	0.095	0.861	0.715	1.037	0.115
PHQ												
Male	0.096	0.096	1.101	0.912	1.328	0.317	0.035	0.133	1.036	0.798	1.345	0.790
Age	− 0.018	0.006	0.982	0.971	0.994	0.003	− 0.019	0.011	0.981	0.961	1.003	0.087
Married	− 0.408	0.215	0.665	0.436	1.014	0.058	− 0.364	0.446	0.695	0.290	1.664	0.414
Education (1–7)	− 0.310	0.069	0.733	0.640	0.840	< 0.001	− 0.082	0.128	0.922	0.717	1.184	0.523
Obesity												
Male	0.307	0.108	1.359	1.101	1.679	0.004	− 0.142	0.111	0.868	0.698	1.079	0.202
Age	− 0.005	0.004	0.995	0.987	1.003	0.187	0.003	0.007	1.003	0.989	1.017	0.676
Married	− 0.002	0.135	0.998	0.766	1.300	0.987	0.070	0.248	1.073	0.659	1.745	0.778
Education (1–7)	− 0.106	0.046	0.899	0.822	0.983	0.020	− 0.111	0.079	0.895	0.767	1.044	0.157
Cigarette smoking												
Male	0.085	0.088	1.089	0.917	1.293	0.332	0.680	0.362	1.973	0.971	4.012	0.060
Age	− 0.019	0.006	0.981	0.971	0.992	0.001	0.009	0.011	1.009	0.988	1.030	0.419
Married	− 0.604	0.204	0.547	0.367	0.815	0.003	− 0.732	0.400	0.481	0.219	1.053	0.067
Education (1–7)	− 0.366	0.065	0.694	0.610	0.788	< 0.001	− 0.331	0.113	0.718	0.575	0.896	0.003
E-cigarette vaping												
Male	0.309	0.140	1.362	1.036	1.791	0.027		-	-	-	-	-
Age	− 0.052	0.009	0.950	0.932	0.967	< 0.001		-	-	-	-	-
Married	− 0.739	0.358	0.477	0.237	0.962	0.039		-	-	-	-	-
Education (1–7)	− 0.400	0.111	0.670	0.540	0.832	< 0.001		-	-	-	-	-

## Discussion

This study demonstrated that, in general, higher educational attainment had favorable associations with cigarette smoking, e-cigarette vaping, CMC, SRH, depressive symptoms, and obesity among American adults. In addition, there was no significant interaction between race and educational attainment on CMC, SRH, depressive symptoms, obesity, cigarette smoking, and e-cigarette vaping during the COVID-19 pandemic. As this is contrary to many observations from studies of MDRs theory in the same data set and other data sets before the COVID-19 pandemic, the COVID-19 pandemic might have played as an equalizer of the health returns of educational attainment for Black and White individuals in the USA.

Consistent with our first finding, educational attainment, as a key SES indicator, has been constantly associated with favorable health and behavioral outcomes across various populations, gender, and age groups [13, 55, 56]. Educational attainment is associated with better mental health [57, 58] and protects against obesity [15] and cigarette smoking [16]. Long-run longitudinal studies in the USA, such as Americans’ Changing Lives (ACL) Study with more than 4000 individuals and 25 years of follow-up [8], Health and Retirement Study (HRS) with a nationally representative sample of more than 35,000 individuals over age 50 who were followed for more than 30 years [59, 60], and Add Health Study with a nationally representative sample of 20,000 students in grades 7–12 in 1994–1995 who have been followed for 15 years through adolescence and the transition to adulthood [61] have all demonstrated that higher levels of educational attainment are associated with lower mortality [62] and morbidity [8]. Similar results are shown from the British Whitehall Study [63] and French GAZEL cohort [64]. For instance, research by Marmot, using the data from Longitudinal Whitehall study, demonstrated that most educated professionals experience the lowest mortality [65, 66]. Similar results replicated for grade of employment, which can be considered as a proxy of educational attainment and income, after 25 years follow-up [67]. Furthermore, assessing the duration of cognitive impairment in HRS from 1992 to 2004, Reuser et al. found that higher educational attainment was associated both with longer life expectancy and shorter time lived with cognitive impairment [68].

Several mechanisms are suggested for why educational attainment can predict health and behavioral outcomes. Individuals with less educational attainment are subjected to greater chronic stress [69]. Individuals with high educational attainment feel in control of their lives, which is enabling for maintaining a healthy lifestyle [70]. Furthermore, educational attainment promotes health by providing a better environment, faster response to health information, and healthy development [16, 71]. Mirowsky and Ross believed that the effect of educational attainment on health is enduring, consistent, and growing over time [70]. In their opinion, education empowers individuals to overturn the default American lifestyle, which prioritize mechanical energy over human energy, industrial food production over household food production, and medical dependency over health maintenance [56]. They proposed three categories of mediators for educational attainment’s beneficial effect on health: (1) work and economic parameters such as income and employment status; (2) psychosocial resources, such as social network and the sense of personal control; and (3) health lifestyle factors, such as smoking status, physical activity, drinking habits, and use of medical services [72]. Education as a *commodity* and education as *human capital* and *learned effectiveness* are two theories, although not mutually exclusive, that have been used by Mirowsky and Ross to explain why education enhances health, each emphasizing various primary links between educational attainment and health [72]. Theories of *learned effectiveness* focus on sense of personal control and health lifestyle as mediators, while *commodity* theories emphasize household income and health insurance and access to medical care as primary mediators [73, 74].

In this study, we observed that, during the COVID-19 pandemic, educational attainment demonstrated a similar associations with health and behavioral outcomes among Black and White individuals. In line with our finding, during the COVID-19 pandemic, Goldmann et al. found that poor mental health indicators were lower among Black individuals compared to White individuals despite the higher prevalence of pandemic-related stressors [45]. There are also comparable findings from studies on previous social and financial adverse incidents in recent US history before the COVID-19 pandemic. For example, during the 2009 financial crisis, researchers showed a significant association between the foreclosure rate and an increase in suicide which was even stronger for White males [46]. Furthermore, after the September 11 Attacks, African Americans demonstrated relatively the same mental health outcomes as White participants [47]. However, a recent study during the COVID-19 pandemic showed persistent diminished health returns of employment in Black individuals compared to White individuals [40].

Our findings show a significant discrepancy from previous MDRs research conducted prior to the COVID-19 pandemic. Contrary to our findings, Black individuals’ diminished returns of educational attainment had been consistently shown for subjective health [28], happiness [75], obesity [27], and cigarette smoking [26]. In addition, Luo et al. demonstrated Black individuals’ diminished returns of educational attainment for sleep disorders [76]. In a cross-sectional study of 10,619 youth aged 12 to 17 years conducted in 2013, before the COVID-19 pandemic, diminished returns of parental educational attainment were found for psychological distress for Black youth compared to White youth [77]. In another cross-sectional study out of the COVID-19 pandemic of 881 American adults conducted using data from the 2017 State of The State Survey (SOSS), diminished returns of income on psychological distress were shown for Black individuals compared to the White individuals [78]. Using the data of 2884 American adults from the Social Justice Sexuality Project (SJS-2010), research found that high educational attainment better protects heterosexual individuals against obesity compared to homosexual individuals [27]. Before the COVID-19 pandemic, in a cross-sectional study using data of 2277 American adults from the Health Information National Trends Survey (HINTS) 2017, smaller protective effect of educational attainment on cigarette smoking was shown in Black individuals compared to White individuals [26]. Discrepancy of our findings during the COVID-19 pandemic from previously established MDRs points toward probable effective forces that equalized health return to educational attainment between racial groups during this pandemic. The following paragraphs discuss the reasons and factors behind equal health returns to educational attainment during the COVID-19 pandemic.

The COVID-19 pandemic was accompanied by severe social restrictions that applied to both privileged and underserved racial groups [79]. Cruise ships were canceled, theatres and gyms were closed, and in-person leisure activities became impossible in this unprecedented period [80, 81]. Because of the initial uncertainty and interruption of established social structure caused by COVID-19, some proposed COVID-19 as the “Great Equalizer” [82], which was doubted later due to the disproportionate death toll and financial burden on racial minorities emerged in the course of the COVID-19 pandemic [83, 84].

The COVID-19 pandemic caused more than half a million excess death in 2021 [34], and death due to COVID-19 was undoubtedly higher in marginalized and underserved populations [85, 86]. This can be attributed to a lack of access to appropriate health care [87], previously worse health conditions [88], and other poor health determinants in racial minorities, all of which can be stemmed from structural racism [89], which in turn, made them more vulnerable to health catastrophes like COVID-19 [90]. Accordingly, Ong et al. demonstrated that 73% of Black individuals in Los Angles inhabit neighborhoods with the highest level of pre-existing health difficulties [88]. Because of these devastating consequences, some would argue that the COVID-19 pandemic increased the class gap or racial discrimination [83]. However, the mental and physical health impacts of a disaster such as COVID-19 can be considered separately from its casualty [45]. We can differentiate between the direct health consequences of COVID-19, such as mortality, and the social and financial crises that ensue from it [91]. Our findings of similar returns of educational attainment for White and Black individuals during the COVID-19 pandemic do not contradict the fact that Black individuals experienced significantly higher mortality rates due to COVID-19.

According to MDRs theory, the diminished returns of SES resources for marginalized groups including Black individuals are due to the racism that acts at multiple levels and through different forms such as structural racism [92], residential segregation [93], discrimination in the workplace [94, 95], poor education quality [96, 97], unsafe neighborhoods, and lack of access to good healthcare [87, 88], preventing Black individuals from obtaining tangible health and financial returns regardless of their SES level [24, 25]. But, it seems that the COVID-19 pandemic, at least in its first months of emergence, diluted the impact of previously unfair social structures that are the cornerstone of racism and MDRs of racial minorities.

MDRs theory uses well established theories of racism to explain the diminished returns of marginalized groups. Racism is defined by Williams and Mohammed as a multi-form phenomenon embedded in American society [92] that through institutional racism, cultural racism, and experienced racial discrimination adversely impacts the health of non-dominant racial populations, and it has systematically reduced access of minorities to housing, employment opportunities, neighborhood and educational quality, and other desirable resources. Gee and Ford have proposed that racism, especially at structural rather than individual levels, is the main origin of the disproportionate burden of morbidity and mortality experienced by racial minorities. Various aspects of structural racism include policies, social segregation, and intergenerational effects that are in charge of racial health disparities in the USA. As such, research and policy should focus on multiple dimensions of structural racism as fundamental cause of health inequities in the USA [98]. Besides, Krieger and Bailey believe that public and private institutions, hand in hand, have maintained racial hierarchies that provided the White Americans across generations with the opportunity to accumulate more wealth and maintain political power than non-White American. In their opinion, this structural racism has contributed to the unfair distribution of social determinants of health and persistent health inequities [99]. Therefore, racism is generally considered the main reason behind MDRs.

The unprecedented circumstances caused by the COVID-19 pandemic might have balanced life conditions for both Black and White individuals. As adversity is required for preparedness, White individuals probably could not effectively mobilize their resources during the COVID-19 pandemic (especially the early months) as they had less experienced social and economic adversities before the COVID-19 pandemic [100]. Following the interruption in healthcare use due to the COVID-19 pandemic [39], it is plausible that the health decline following the COVID-19 pandemic could be smaller for racial minority individuals who already had poor access to healthcare prior to the COVID-19 pandemic [92, 101, 102]. Previous research before the COVID-19 pandemic had shown that individual poverty, neighborhood poverty, and Black race were associated with lower odds of physical activity [103]. Black individuals who already had lower odds of physical activity would be less affected by the closure of gym and fitness centers compared to White individuals who could better afford using such resources. This is supported by the observation that those with the highest levels of physical activity before COVID-19 reported the sharpest declines in their physical activity during the COVID-19 pandemic [104]. Hence, the disruption of the previously established social order could differently influence different racial groups: While crises do not discriminate, at least in some respects, some groups may be less prepared for such disruption. The COVID-19 pandemic, just like the 2009 financial crisis and September 11 Attacks, probably put individuals in a situation, even temporarily, in which the privileged cannot benefit from their privilege. In this view, Whites could become vulnerable because of losing their relative advantage, resulting in comparable outcomes for all racial groups after these catastrophes [45–47].

In addition, we know that each additional risk factor interacts with present stressors and resources. In general, the interaction between the adverse effects of a disaster with individuals’ pre-existing life stressors can be sub-additive or additive (cumulative) [105]. If the additional stress of the COVID-19 pandemic had a cumulative effect, Black individuals should have shown a larger decline in mental health and behavioral outcomes in the wake of adverse events. In fact, should the stressors be added cumulatively to the existing risk factor, we should have seen a considerable increase in the incidence of mental and physical problems in marginalized racial groups in the wake of the COVID-19 pandemic, the 2009 financial crisis, and the September 11 Attack [47]. Results from the COVID-19 pandemic, September 11 Attacks, and the 2009 financial crisis do not support such assumption [45–47]. Given that the interaction of psychiatric comorbidities on suicide is sub-additive, not cumulative/synergistic [105], we can postulate Black individuals who already had tolerated significant distress did not show a similar increase in negative health and behavioral outcomes in response to the COVID-19 pandemic as White individuals. In this regard, because of the Black individuals’ pre-existing high level of stressors and sub-additive nature of additional stressors the final burden tended to be comparable between White and Black individuals in previous national disasters [106]. This does not deny higher mortality of Black individuals from COVID-19 [85, 86]. However, among those who survived and participated in the HINTS 2020 survey, White individuals’ wellbeing may have been affected more adversely than Black individuals.

Black individuals’ resilience in the face of adversity has been articulated in detail in Black-White Paradox’s in mental health theory [107]. According to this theory, Black individuals develop mental resilience in response to greater social inequality, racial discrimination, and high rates of physical morbidity [107–109]. They have lower rates of common mental disorders and higher rates of flourishing compared to White individuals [107], maintaining their mental health despite adverse conditions. Based on the definition, to be diagnosed as flourishing in life is as follows: “individuals must exhibit high levels on at least one measure of hedonic wellbeing and high levels on at least six measures of positive functioning” [110]. Flourishing has been shown to be adaptive for Black and White individuals in terms of three important indexes of burden to society: depression, work loss, and disability [107]. Erving et al. provided more evidence that Black individuals had lower or similar rates of mental disorders across lifetime and past-year using data from the National Comorbidity Survey Replication and the National Survey of American Life, 2001–2003 [108]. This theory can explain Black individuals’ resilience despite higher death tolls and worse financial conditions than White individuals during the COVID-19 pandemic. In other words, Black individuals who had already experienced adverse life conditions before COVID-19 had become more resilient to COVID-19’s devastating effects. In this regard, resilience is a gift of adversity, and vulnerability is the cost of privilege [25].

Our findings during the COVID-19 pandemic suggest that social factors are responsible for Black individuals’ diminished return of educational attainment [111]. The COVID-19 pandemic provided a unique glimpse into the social conditions when advantaged White individuals cannot mobilize their resources as usual [112]. Under the same social circumstances, both White and Black individuals will act almost the same and produce comparable findings. Thus, this study supports our claims that the observed diminished return of educational attainment for Black individuals is due to social situations, particularly structural racism, not biological causes [113, 114].

## Implication

Our study has implications for researchers and policymakers. The COVID-19 pandemic provided a rare opportunity for researchers to study how the dynamic of social forces changes during unexpected macro events. We used several theories to explain the underlying mechanisms responsible for racial disparity or class gaps and what social factors may alleviate them. Among those who survived the COVID-19 pandemic, the association between educational attainment and CMC, SRH, depressive symptoms, obesity, cigarette smoking, and e-cigarette vaping are not larger for Black individuals than White individuals. Our preliminary findings pave the way for future research on how the COVID-19 pandemic might change racial disparities in our society for the better.

Policymakers should also be mindful of the impact of the COVID-19 pandemic on the physical and mental health across various racial groups. Considering that the COVID-19 pandemic, at least for outcomes studied here, seems to be an equalizer for the returns of educational attainment among racial groups, policymakers can adopt similar strategies to ones implemented during the COVID-19 pandemic, such as financial support (e.g., sending paychecks) [115] and providing housing (e.g., protecting renters) [116], to help Black individuals mobilize their SES resources to improve their health. While before the COVID-19 pandemic free market was the main factor in the US society, during the COVID-19 pandemic, governmental interventions were more prominent [117]. Given the diminished returns of educational attainment before the COVID-19 pandemic, one can infer that governmental intervention such as availability of cash, financial assistance, affordable housing, and accessible healthcare probably contributed to better outcomes regarding the health returns of SES for racial minorities.

Traditional approaches toward racial health disparities mostly focused on equality of access to SES resources as a means to reduce racial health disparities [118, 119]. From this perspective, marginalized individuals will have comparable health and financial outcomes to their White counterparts, provided they have similar SES resources. On the contrary, research in the MDRs field usually implies that social policy should go beyond equality in access. As it is challenging and complicated and even impossible to do a social experiment at a national level, we considered COVID as a unique opportunity to find out what factors affect the diminished returns of marginalized individuals. It seems that the decrease in financial freedom [120], in combination with the unequal distribution of aid in a way that benefited mostly those at the bottom of the income pyramid [121], led to no significant difference in the health return of educational attainment for Black and White individuals. If the same pattern repeats over longer periods during the COVID-19 pandemic in future studies, it can be assumed implementing similar strategies and direct financial support can be a potential measure to help marginalized individuals mobilize their SES resources. Our findings, especially if resulted solely from direct government support, contradict the previous outcomes in the MDRs field, which repeatedly highlighted that equal access to SES resources could not completely reduce racial health disparities [122].

Still, one interesting future line of research would be to determine the differential effect of disruption in the free market and direct government support on MDRs. A study on the effects of a support package designed similarly to diverse government support during the COVID-19 pandemic, including COVID-19 Rental Assistance, COVID-19 Mortgage Relief, COVID-19 Student Loan Forbearance, COVID-19 Stimulus Checks for Individuals [123, 124], being implemented during a normal time can provide us empirical evidence whether equality to access ( monetary aid, housing), can reduce racial health disparities or temporary disruption of structural barriers integrated into the free market system plays at least a part in comparable health return of educational attainment for White and Black individuals.

## Limitation

This study had several limitations. First, causal relations cannot be interpreted from the observed associations because of the observational nature of this study. Second, the COVID-19 pandemic could have distinct impacts on different age and gender groups, which was beyond this study’s scope. Further research is needed to shed light on how these groups, especially children and the elderly, have been affected by the COVID-19 pandemic. Third, we cannot comment on the durability of the observed changes in the racial groups’ health return of SES. Future research can determine whether the COVID-19 pandemic had a long-term impact on US society and whether our results can be replicated. Fourth, considering the limited time period and sample size of the present study, future studies with larger sample sizes, higher response rates, and over longer periods during COVID-19 pandemic, preferably in other datasets such as US Census Bureau’s Household Pulse Study (HPS), are warranted.

## Conclusion

In summary, our study was one of the first to assess the effect of COVID-19 as a major social adversity on the interaction between race and educational attainment on several health and behavioral outcomes. The COVID-19 pandemic seems to equalize the health return of education among Black and White individuals through mechanisms explained by nullifying the privilege, Black-White Mental Paradox, and sub-additive interaction of additional stressors. Our findings support the notion that social conditions, not intrinsic biological differences, explain the racial disparities in health and behavioral outcomes and the returns of SES.

## Declarations

### Animal Study

NA
